# 0729. Interstitial changes in spleen during sepsis

**DOI:** 10.1186/2197-425X-2-S1-P51

**Published:** 2014-09-26

**Authors:** ØS Svendsen, L Stangeland, B Elvevoll, BT Gjertsen, J Skavland, H Wiig, O Tenstad, P Husby

**Affiliations:** Department of Anaesthesia and Intensive Care, Haukeland University Hospital, Bergen, Norway; Department of Clinical Science, University of Bergen, Bergen, Norway; Department of Clinical Medicine, University of Bergen, Bergen, Norway; Department of Biomedicine, University of Bergen, Bergen, Norway

## Introduction

The spleen has important functions in innate and acquired immunological responses, and splenectomy leads to risk for overwhelming infections [1]. The microcirculation in spleen is only partly understood, and knowledge of the local interstitial fluid composition and changes during sepsis can offer new insights in the systemic inflammatory response syndrome.

## Objectives

To isolate prenodal lymph from spleen in pigs during a control situation and during sepsis, and analyze the composition of proteins in the lymph.

## Methods

Pigs were anesthetized and monitored. Plasma volumes were measured and the changes in hematocrit were, after corrected for the fluid gain and loss, used to estimate global plasma extravasation. One pre-nodal spleen lymph-vessel was cannulated. LPS was administered to induce sepsis. Lymph and plasma were studied to investigate the specific local reaction in spleen during control situation and sepsis.

## Results

Basal extravasation rate was 0.24±0.05 mL/kg*min^-1^(n=4). Although technically demanding, we succeeded in collecting lymph from the spleen, deemed macroscopically to be prenodal. After inducing sepsis, pulmonary artery pressure increased from 19.0±2.2 mmHg to 30.4±4.6 mmHg one hour after LPS, (n=5, p=0.001). Adrenaline and Ringer's acetate were administrated to support the animals through the inflammatory response. Plasma extravasation rate increased twofold to 0.53±0.04 mL/kg*min^-1^ (n=4, p=0.003). Colloidosmotic pressure in plasma was 14.1±0.6 mmHg in control situation, decreasing to 12.9±0.5 mmHg (n=4, p=0.003) one hour after LPS. Colloidosmotic pressure in lymph was 13.0±1.7 mmHg (n=4) in control situation. During the first hours after sepsis, colloidosmotic pressure in spleen lymph decreased to 11.8±2.4 mmHg (n=3).

## Conclusions

Our model shows typically hemodynamic changes previously described to be associated with an initial endotoxin reaction in pigs [2]. We show that it is possible to isolate lymph from spleen. The colloidosmotic pressures in lymph and plasma suggest a low protein reflection coefficient in spleen microvasculature.
